# Organ-specific isogenic metastatic breast cancer cell lines exhibit distinct Raman spectral signatures and metabolomes

**DOI:** 10.18632/oncotarget.14865

**Published:** 2017-01-27

**Authors:** Paul T. Winnard, Chi Zhang, Farhad Vesuna, Jeon Woong Kang, Jonah Garry, Ramachandra Rao Dasari, Ishan Barman, Venu Raman

**Affiliations:** ^1^ Department of Radiology and Radiological Science, Johns Hopkins University School of Medicine, Baltimore, MD 21205, USA; ^2^ Department of Oncology, Johns Hopkins University School of Medicine, Baltimore, MD 21205, USA; ^3^ Department of Pathology, University Medical Center Utrecht Cancer Center, 3508 GA Utrecht, The Netherlands; ^4^ The Johns Hopkins University, Department of Mechanical Engineering, Whiting School of Engineering, Baltimore, MD 21218, USA; ^5^ Laser Biomedical Research Center, Massachusetts Institute of Technology, Cambridge, MA 02139, USA

**Keywords:** raman spectroscopy, breast cancer, isogenic cell lines, metastases, biochemical signatures

## Abstract

Molecular characterization of organ-specific metastatic lesions, which distinguish them from the primary tumor, will provide a better understanding of tissue specific adaptations that regulate metastatic progression. Using an orthotopic xenograft model, we have isolated isogenic metastatic human breast cancer cell lines directly from organ explants that are phenotypically distinct from the primary tumor cell line. Label-free Raman spectroscopy was used and informative spectral bands were ascertained as differentiators of organ-specific metastases as opposed to the presence of a single universal marker. Decision algorithms derived from the Raman spectra unambiguously identified these isogenic cell lines as unique biological entities – a finding reinforced through metabolomic analyses that indicated tissue of origin metabolite distinctions between the cell lines. Notably, complementarity of the metabolomics and Raman datasets was found. Our findings provide evidence that metastatic spread generates tissue-specific adaptations at the molecular level within cancer cells, which can be differentiated with Raman spectroscopy.

## INTRODUCTION

Breast cancer is the most common malignant neoplasm and is the second leading cause of cancer-related death among women in the United States, exceeded only by lung cancer [1]. The American Cancer Society recently reporting a 5-year survival rate near 99% for local breast cancer [1, 2]. However, the 5-year survival for metastatic breast cancer that involves distant organs drops to a dismal 24% [1, 2]. This situation persists because understanding the metastatic progression of breast cancer remains challenging. This is due to several factors including a limited predictability as to which primary tumor is prone to metastatic progression, an inability to monitor the onset of successful metastatic growth, and incomplete knowledge of metabolic, physiologic, and molecular adaptations that allow for the cancer to survive and thrive within the different tissue types [3]. As such, procuring safe and efficacious chemotherapeutic regimen strategies that ablate metastatic lesions is an unmet clinical need [4]. In addition, the current practice of systemic administration of cytotoxic chemotherapy is limited with respect to targeting and drug resistance, which results in numerous adverse side-effects and no cures [5].

When considering potential solutions to this problem an important factor is the divergence of the metastatic cancer cells growing in visceral organs from the primary breast tumor cells [6–18]. Thus, there is a growing consensus from retrospective as well as prospective clinical trials that matched primary breast tumor and metastatic lesion biopsy samples often exhibit divergent expression of established biomarkers, for example, ER and HER2 [7, 9–11, 17]. Therefore, metastatic lesions should not be considered simply as primary tumor implants at new sites but instead as significantly divergent tissue-specific lesions, which reflect adaptations to organ-specific environments [18]. Importantly, it is very difficult to discern if various organ-specific metastatic lesions will have similar sensitivity to prescribed therapeutic regimens. Accordingly, the organ-specificity of the metastatic spread needs critical reconsideration, as, at present, databases of molecular profiles of matched primary and metastatic breast tumors have not been compiled to address global distinctions between metastatic sites and thus, cannot facilitate generalized metastatic site-specific nor patient specific smart therapeutic alternatives. Consequently, present clinical treatment decision options for distant metastatic breast cancer that rely on an evaluation of a few select biomarkers found during assessment of the primary tumor, although beneficial to subpopulations of patients [19], is also a likely contributing factor to the overall diminished response rates for survival from metastatic disease [9–11, 13]. Such a conclusion is in line with the reported presence of altered and distinct biomarker signatures of the metastatic lesions with respect to those found at the corresponding primary tumor [7, 9–11, 17, 18], which, when evaluated, may indicate that a change in an ongoing treatment strategy should be considered.

Dissecting metastatic cancers based on objective molecular markers remains an important challenge. Here, we propose a fundamentally different approach towards identification of defining metastatic cancer cell signatures from those of primary tumor cells. Harnessing the exquisite specificity of Raman spectroscopy in detecting molecular phenotypes in cells and tissue, we aimed to obtain rapid and label-free profiling of newly generated isogenic metastatic human breast cancer cell lines, which were produced from a xenograft mouse model. Given its lack of sample preparation requirements and ability to provide quantitative biochemical analyses in near real-time conditions, Raman spectroscopy provides a powerful tool for live cell analysis [20]. While this spectroscopic technique has been recently used to distinguish between, normal, benign, and malignant breast tissues, by us and others [21–24], the potential for using these spectral markers as new routes to recognition of metastatic cell types that are isogenic to the primary tumor, as is the clinical case, has been understudied.

Starting from an orthotopic xenograft based mouse model system, the studied human cell lines were obtained from cultured organ-of-origin explants of: brain, liver, lung, and spine, as well as from the primary, i.e., mammary fat pad (MFP), site. These metastatic sites are representative of the common clinically observed breast cancer metastatic destinations [25, 26] with spine representative of bone. Despite being isogenic, these cell lines exhibit important morphological and growth distinctions that support our hypothesis that each metastatic site imbues metastatic tumors with unique molecular attributes. Our Raman spectroscopic measurements reveal the presence of consistent spectral differences of the cell lines. Using multivariate chemometric methods, we show that these spectral changes can be utilized to develop decision algorithms with high diagnostic power. Furthermore, we identify the presence of spectrally informative features that bring to light each cell line's unique spectral characteristics, which reflect the inherent biochemical distinctions. We reason that these differences are a result of intricate reciprocal interactions between the cancer cells, parenchyma, and stroma at the target organ during metastatic growth. Combined with the ability to assay the stromal features, our findings underscore the relevance of Raman spectral information in characterizing isogenic metastatic lesions at different sites in terms of inherent biochemical determinants without staining or requiring *a priori* knowledge of the molecular transformations. In addition, preliminary metabolomic analyses provide supporting data indicating that cancer cells from different metastatic sites acquire metabolic changes, which may define a cell line's metastatic organ of origin. Notably, a complementary overlap between the metabolite distinction data set and Raman spectroscopic signatures was also found.

## RESULTS

### Isogenic metastatic breast cancer cell lines from specific organs

**Figure 1:**
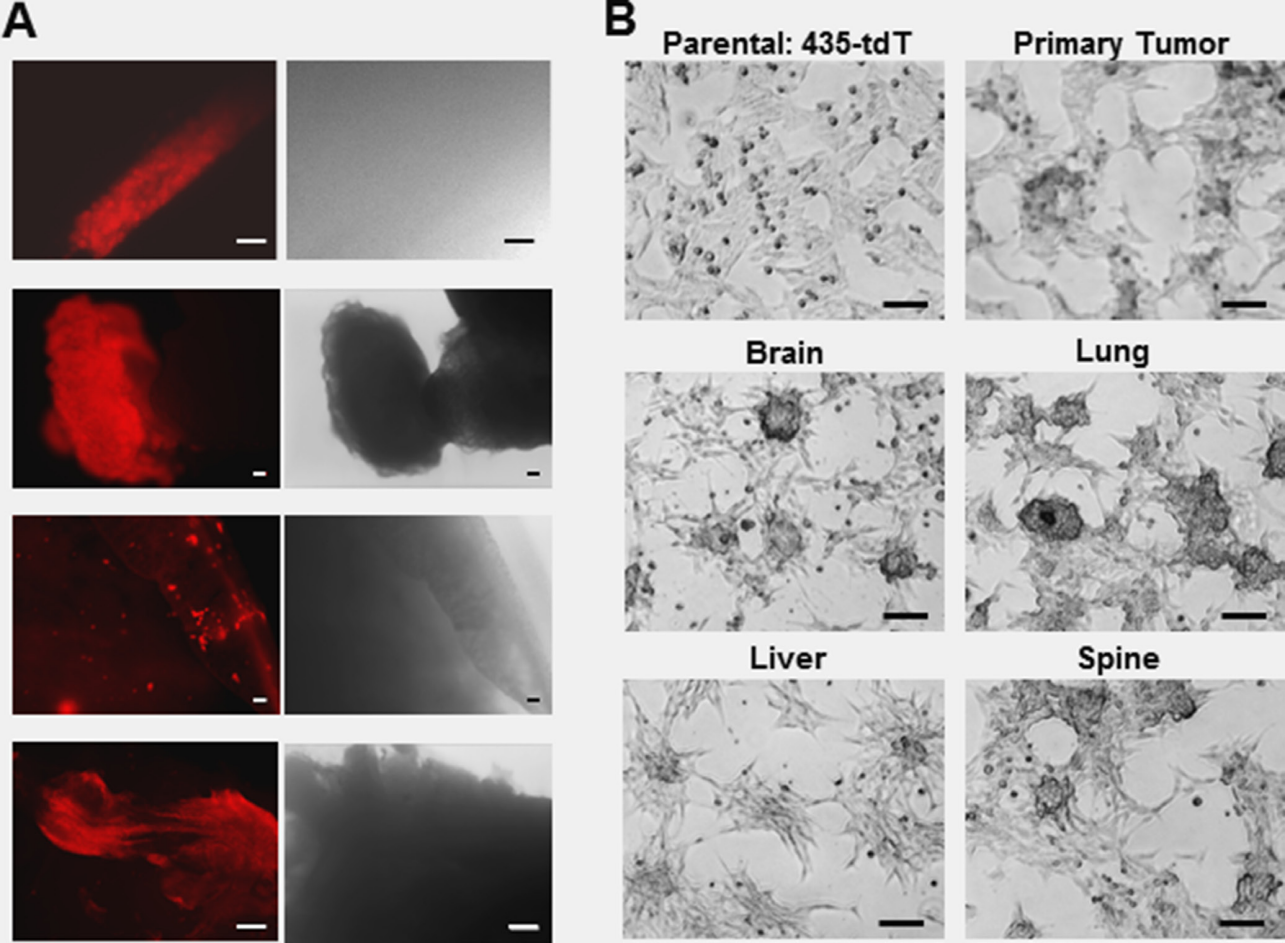
Use of fluorescent microscopy to assess the locations of metastatic lesions in *ex vivo* organ samples and the growth patterns of the subsequent pure metastatic cell lines (**A**) Fluorescence and corresponding phase-contrast images of brain, liver, lung, and spine tissue explants immediately after dissection. (**B**) Phase contrast images of the different colony growth patterns of pure brain, liver, lung, and spine metastatic sublines as well as the primary tumor cell line, compared to the monolayer growth pattern of parental 435-tdT cells. Microscopy was on a Nikon eclipse TS100 inverted microscope using in (A) a 10× objective for brain and spine or 4x objective for liver and lung images, while all images in (B) were obtained using a 10× objective. Microphotographs were acquired using a Roper Scientific CoolSnap™ ES camera, images were captured with NIS-Elements F3.2 software, and processed with ImageJ. Scale bars in all images depict 100 μm.

In order to facilitate the tracking of metastatic progression in live mice [27], we engineered triple negative MDA-MB-435 human breast cancer cells [28–34] to stably express a red fluorescence protein (tdTomato) and here designate this cell line: 435-tdT. Using 435-tdT cells, we initiated the culturing of new organ specific metastatic breast cancer cells (Figure 1A and 1B) with the inoculation of 435-tdT cells into the second thoracic mammary fat pads of female NOD-SCID mice. Phase-contrast images of fresh organ explants showed unresolved amorphous material without evidence of metastatic lesions while the bright tdT-fluorescence revealed the presence of the cancer (Figure 1A). Identified metastatic lesions were placed into cell culture and metastatic cancer cells grew out of native tissue environments until pure populations of red fluorescent cancer cells were obtained.

**Figure 2:**
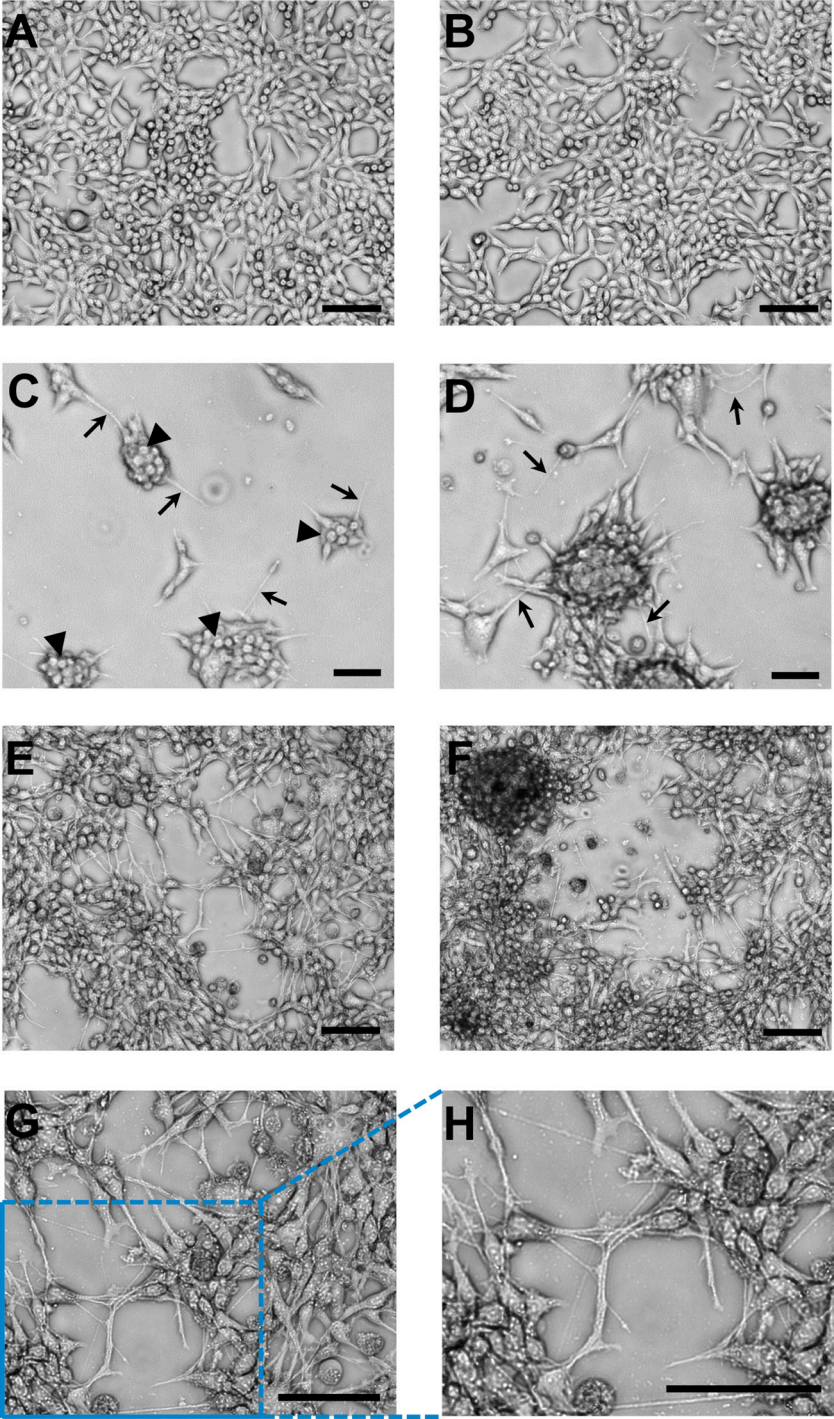
Representative images of the brain cell line growth patterns on adherent plastic compared to monolayer growth of the parental cell line (**A**–**B**) Two fields-of-view of characteristic monolayer growth of the parental cell line. (**C**) Distinct separate colony growth was apparent at 48 hr post inoculation of the plate with distinct small spherical cells making up each colony (arrowheads) and thin cellular extensions/filopodia (micro- or nanotubes; arrows). (**D**) After 120 hr the interconnected colony pattern remained. (**E**–**F**) Two examples of the characteristic growth pattern at “confluency” of the brain cell line with colonies elaborately linked together by nanotubes. These interconnections between cells/colonies have consistently been recorded at > 100 μm in length (see scale bars). (**G**) Higher magnification of the central portion of image (**E**). (**H**) Expanded image of the lower left-hand corner of image (**G**). These magnified images allow for a very clear visualization of the complex and intricate web of interconnections between colonies that were in place. Microscopy was on a Nikon eclipse TS100 inverted microscope using for (**A**–**F**) a 10x objective and for (**G**) a 20× objective. Both objectives were used in combination with a 4× phase contrast ring, which produced the high resolution 3D-like images. Microphotographs were acquired using a Roper Scientific CoolSnap™ ES camera, images were captured with NIS-Elements F3.2 software, and processed with ImageJ. Scale bars in all images depict 100 μm.

Once adapted to plastic, all metastatic cell lines as well as the primary tumor cells grew as loosely adherent 3D spherical colonies made up of tightly packed spherical cells with various degrees of monolayer growth (Figure 1B), which is starkly different from the overall monolayer growth of the parental 435-tdT cell line (Figure 1B and Figure 2A and 2B).

**Figure 3:**
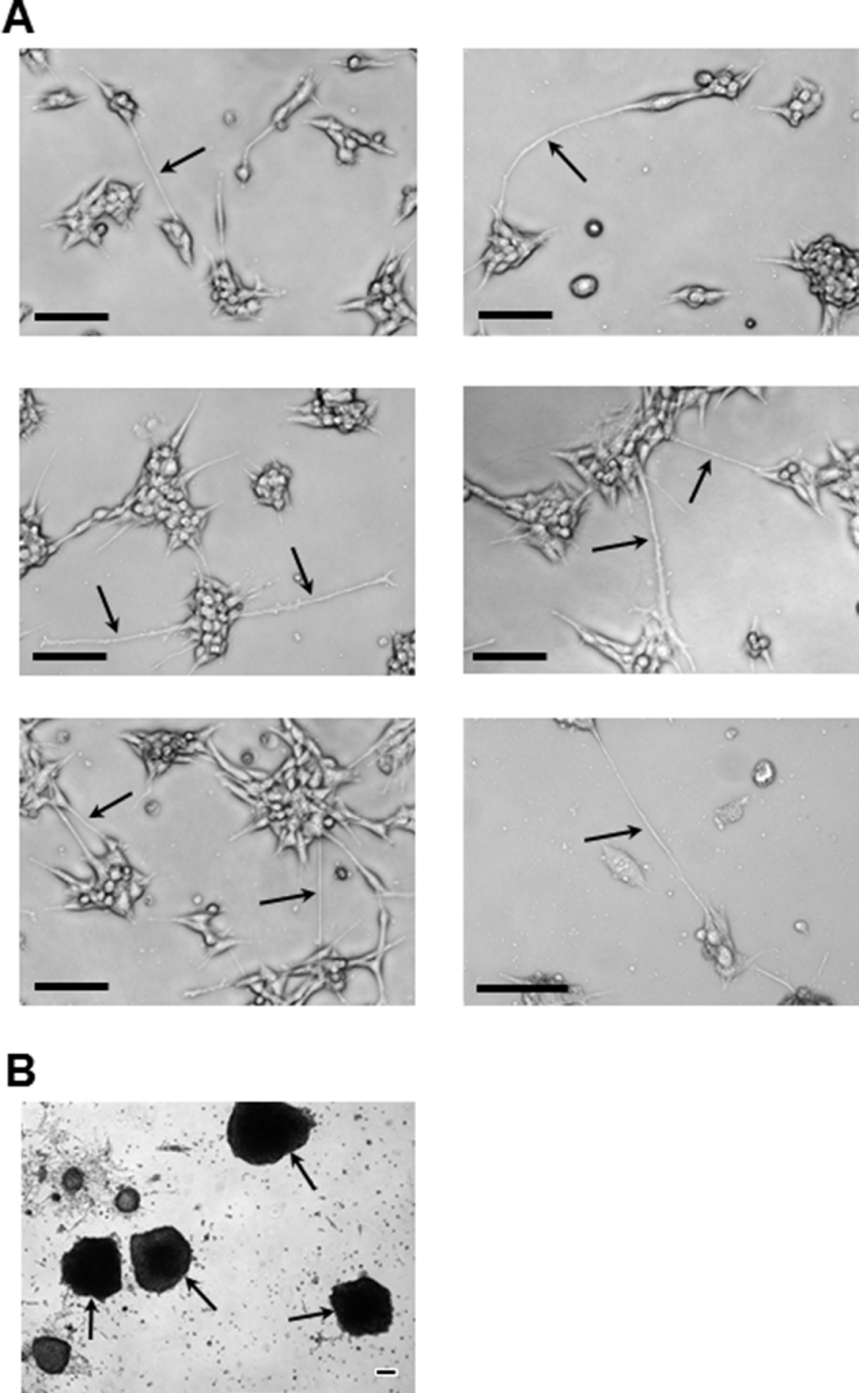
Representative images of the brain cell line colony and mammosphere growth patterns (**A**) Images highlighting (arrows) the very long (> 100 μm: see scale bars) nanotube interconnections (or filopodia; e.g., middle right-hand image) that consistently form during: 24 hr (top row), 48 hr (middle row), and 120 hr (bottom row) of growth. (**B**) Examples, under adherent culture conditions, of the large free-floating mammospheres (arrows) that consistently formed during subculturing of smaller floating mammospheres retrieved from confluent brain cell line culture medium. All imaging equipment and image processing was the same as described in Figure 1. All images in (**A**) were obtained with a 10x objective. The image in (**B**) was obtained using a 4x objective. Scale bars in all images depict 100 μm.

Representative images exemplified by brain cell line growth patterns show the brain cell line had a colony growth pattern as contrasted to the monolayer growth of the parental cell line (Figures 2 and 3). Colonies were apparent as early as 24–48 hr post seeding of (Figure 2C–2D and Figure 3A-top and middle images). Distinct small spherical cells making up each colony are readily seen (Figure 2C) and this growth pattern remains throughout culturing as exemplified at 120 hr of growth (Figure 3A-bottom images). In addition, thin cellular extensions, micro- or nanotubes, (Figure 2C–2D and Figure 3A) were visible, which at relatively low cell numbers, i.e., at 24–48 hr, appeared to be attached to the substratum and also as connections between adjacent colonies. These connections become more numerous as the cultures grew (Figure 2D and Figure 3A). A characteristic pattern at “confluency” (Figure 2E–2F) with colonies elaborately linked together by nanotubes is contrasted to monolayer growth patterns of the parental cell line (Figure 2A–2B). Interconnections between cells/colonies have consistently been recorded at > 100 mm in length (Figure 2C–2H and Figure 3A) when connecting distant cells/colonies. At high cell/colony numbers, depicted in two fields-of-view (Figure 2E–2F) and in magnified and expanded images (Figure 2G–2H), show that a complex and intricate web of interconnections between colonies often occurred. In addition, free floating small and large mammospheres (Figure 3B) and small floating colonies along with free floating single cells (most abundant in brain and spine cell cultures) were a consistent feature of these cultures, which was unexpected under the adherent plate conditions used throughout the culturing process.

Overall, it was apparent that important distinctions between phenotypes and growth patterns (Figure 3) were present between cell lines. This and the very distinct non-monolayer growth, ultimately reflect genetic diversity following metastasis and adaptation to the surrounding unique tissue environments.

### Isogenic metastatic breast cancer cell lines: average specific growth rates

**Figure 4:**
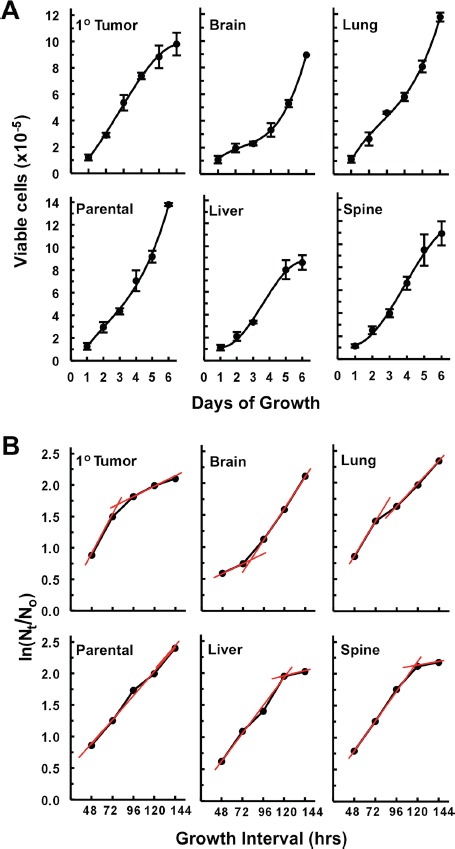
Growth curves and estimation of average specific growth rates (μ) off of plots of ln(N_t_/N_o_) versus time (**A**) Growth curves of viable cell numbers vs days of growth depicting distinctions in growth characteristics between cell lines. Each data point of the growth curves represents a mean (*n* = 3 to 4 wells of cells) ± 1 standard deviation (error bars) except for the last point of the brain and the last two points of the liver where these are averages of two wells of cells. (**B**) The same data sets use in (A) plotted as ln(N_t_/N_o_) vs growth interval in hrs where N_t_ is the number of cells at time ‘t’, N_o_ is the initial number of cells, i.e., viable cell counts on day 1 (24 hrs after seeding the plates), and t is time. As, ln (N_t_/N_o_) = μt, it can be seen that the slope (μ) of each treadline (shown in red) provides an estimate of the average specific growth rates over the course of each growth interval (red lines) shown (see Materials and Methods for a definition of growth interval).

Average specific growth rates along with average cell cycle times (Table 1) were estimated by linear transformation [35] of the data used to generate the viable cell numbers versus days of growth curves presented in Figure 4A. Qualitative differences between the growth characteristics of the different cell lines can be ascertained from Figure 4A. Thus, it was apparent that primary tumor, liver, and spine cell lines appeared to have little or no lag-phase to their growth, followed by a relative steady rapid growth that ended with a plateau phase of slowed growth and the latter, for the liver cell line, occurred at a relatively low cell density. On the other hand, the brain cell line exhibited a protracted lag-phase that was followed by a rapid growth phase that did not reach a slowing of growth during the time period of this experiment. The lung cell line grew without a lag-phase, passed through a slowing of growth and then rapidly grew until the end of the experiment. The parental cell line had a relatively steady growth rate for most of the time that may have increased somewhat prior to the end of the experiment. The analysis of the plots shown in Figure 4B shed light on these qualitative evaluations. Thus, except for the parental cell line, all cell lines can be evaluated as having two distinct average specific growth rate periods shown as red tread lines in Figure 4B. The slopes of these lines provide estimates of average specific growth rates (μ) for each time period (Table 1), which then allows for the calculation of the average length of the cell cycle (t_c_) for each period (Table 1). As such, it was found that the parental cell line had an average specific growth rate of about 38% per day and a corresponding average cell cycle length of 1.8 days throughout the six days of growth. For the brain, lung, and primary tumor cell lines the two periods of growth were from 24–72 hrs and 72–144 hrs (Figure 4B). While lung and primary tumor cell lines grew rapidly during the first time period, i.e., at average specific growth rates of 55% and 61% per day respectively (Table 1), with corresponding short average cell cycle times of about 1.25 and 1.13 days respectively (Table 1), the brain cell line exhibited a prolonged initial (24–72 hrs) slow average specific growth rate of only about 15% per day (Table 1) that corresponded to an average cell cycle time of 4.65 days. However, between 72 and 144 hrs the brain cell line's average specific growth rate increased greater-than 3 fold to about 50% per day with an average cell cycle time of 1.4 days (Table 1). For the liver and spine cell lines the two periods of growth were from 24–72 hrs and 96–144 hrs (Figure 4B). These cell lines had similar growth characteristics with average specific growth rates of 43 and 46% per day respectively and corresponding average cell cycle lengths of 1.6 and 1.5 days respectively during the first time period (Table 1). Similarly, both liver and spine cell lines reached a stationary growth phase (96–144 hrs) (Figure 3B) where average specific growth rates of only 7.6 and 6.2% per day and corresponding average cell cycle times that increased by nearly 10 fold to about 9 and 11 days respectively (Table 1).

**Table 1:** Average specific growth rates and length of cell cycle divisions for the indicated growth periods

		μ† (Day-1)	μ (hr-1)	tc‡, (Days)	tc (hr)
**Brain**	**24–72 hr**	0.149	0.0062	4.65	111.8
	**72–144 hr**	0.495	0.0206	1.40	33.6
**Parental**	**24–144 hr**	0.383	0.0160	1.81	43.4
**Liver**	**24–120 hr**	0.430	0.0179	1.61	38.2
	**96–144 hr**	0.076	0.0032	9.08	218.0
**Lung**	**24–72 hr**	0.555	0.0231	1.25	30.0
	**72–144 hr**	0.354	0.0148	1.96	47.0
**Spine**	**24–120 hr**	0.464	0.0193	1.49	35.8
	**96–144 hr**	0.062	0.0026	11.18	268.3
**1^o^Tumor**	**24–72 hr**	0.613	0.0255	1.13	27.2
	**72–144 hr**	0.142	0.0059	4.89	117.3

^†^Denotes average specific growth rate from equation: ln(N_t_/N_o_) = μt, where N_t_ is the number of cells at time ‘t’, N_o_ is the initial number of cells, and t is time.

^‡^Denotes the average length of the cell cycle division and is derived from: t_c_ = ln2/μ.

Overall, under these *in vitro* conditions, these results indicate that each of these isogenic cell lines modulates its cell cycle rate along with cell loss or senescence rate and hence its growth rate differently throughout the six-day culture period.

### Isogenic metastatic breast cancer cell lines: motility

Two independent motility assays were carried out in standard 24 well Transwell^®^ plates with 8 mm membrane inserts. During day two (white bars) and three (gray bars), the parental cell line's motility was relatively high (Supplementary Figure 1) as compared to the isolated isogenic cell lines (Supplementary Figure 1). On day two, the motility of the parental cell line was significantly higher than the primary tumor and all metastatic cell lines (*P* < 0.05, two tailed *t*-test), and this remained the case on day three for all cell lines except the liver cell line. By day three the liver cell line's motility was significantly higher than the primary tumor and metastatic cell lines (*P* < 0.005) but not the parental cell line. In addition, it was noted that, except for the parental cell line, the numbers of cells migrating were very low being on average only 1.5% of the total cell numbers in the wells. Also, little or no motility was seen in a “wound”/scratch motility assay (not shown). Both results are consistent with the fact that these metastatic cell lines do not exhibit extensive lateral monolayer growth patterns (Figures 1, 2, 3), which favors migration to and through a pore or into a “wound” but instead propagate vertically in stationary colonies.

### Isogenic metastatic breast cancer cell lines: metabolomics

**Figure 5:**
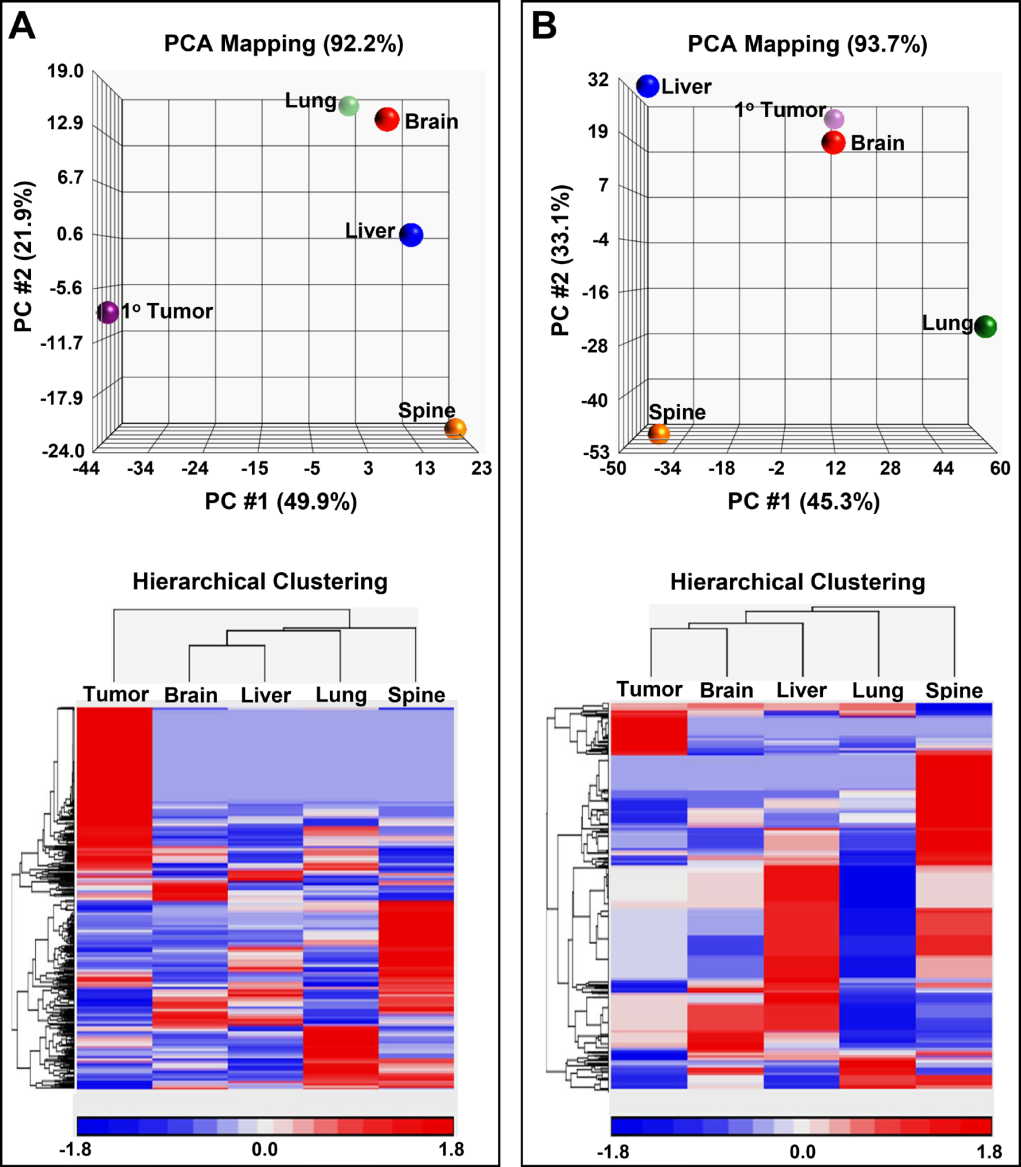
Principal component analysis (PCA) maps along with hierarchical clustering's of metabolites and lipids (**A**) 3D PCA mapping of aqueous metabolites (top panel) displaying sample classes as spheres. Bottom panel displays hierarchical clustering of the samples along with the associated heat map of aqueous metabolite distributions. (**B**) 3D PCA mapping of lipid soluble metabolites (top panel) with spheres representing the sample classes. Panel at the bottom displays a heat map of lipid soluble metabolite distributions along with the associated dendrogram. Expression values for the heat maps are indicated by a key at the bottom of the maps.

To gain a better understanding of the underlying molecular changes that are contributing to or are the result of adaption to different tissue microenvironments metabolomic analyses of our isogenic metastatic breast cancer cell lines were carried out. Our global metabolomics analyses provided strong evidence that our isogenic metastatic cell lines have distinct metabolomes (Figure 5 and Table 2). Principal components analyses (PCA) of aqueous phase as well as lipid phase (predominately lipids) metabolites (Figure 5A–5B) revealed that all the isogenic cell lines unambiguously clustered into discrete classes (depicted as spheres) in both cases, which reflects each cell line's inherently distinct metabolome and lipid characteristics. PCA mapping of aqueous phase metabolites (Figure 5A) shows that PC #1 contributes to 49.9 % and PC #2 contributes to 21.9 %, while PC #3 contributes to 20.4 % (data not shown) of the variation observed in the various samples. PCA demonstrates that there is a large difference, primarily indicated by PC #1, in metabolites of the metastatic cell lines relative to the primary tumor cell line (Figure 5A). Similarly, metastases are mainly differentiated by PC #2 indicating that they are more similar to one another. Hierarchical clustering confirmed that the primary tumor cell line was clustered separately from the metastases (Figure 5C). The dendrogram also confirmed that brain and liver metastases were more related to each other with respect to aqueous metabolite components (Figure 5C). PCA mapping of lipid phase metabolites (Figure 4B) shows that PC #1 contributed to 45.3 % and PC #2 contributed to 33.1 % while PC #3 contributed to 15.3 % (data not shown) of the total variation. PCA showed that primary tumor lipids phase metabolites were closely related to brain lipid phase metabolites. Hierarchical clustering confirmed PCA analysis showing that lipid phase metabolites in primary tumor were closely related to brain (Figure 5D). The largest variation in lipid phase metabolites expression was observed in spine metastases.

**Table 2:** Fold increases of top metabolites from the 1^o^ Tumor^1^ and metastatic sites

Metabolite	1^o^ Tumor vs:			
	Brain	Liver	Lung	Spine
Biotripyrrin	4.2	14.0	7.5	34.6
2-Amino-3-Carboxymuconic Acid Semialdehyde	17.3	14.7	2.9	12.0
L-Thyroxine	----^2^	18.0	2.5	7.9
Phosphatidylinositol Trisphosphate (PIP3) (18:0/16:1)	3.5	4.6	6.7	3.9
Lysophosphatidylethanolamine (LysoPE) (15:0/0:0)	5.4	3.7	2.3	3.7
L-Dihydroorotic Acid	4.0	3.8	2.0	3.6
Cholesterol Ester (14:1)	2.7	3.3	4.8	3.7
Cholesterol Ester (20:4)	2.0	2.2	2.7	3.4
	**Brain vs:**			
	**1^o^ Tumor**	**Liver**	**Lung**	**Spine**
Neurotensin 1-10	85	3.8	100	9.9
Tryptophyl-Tryptophan	60	24	----	36
Phosphotidylethanolamine (PE) (18:3/14:1)	23	2.2	----	9.6
Phosphotidylglycerolphosphate (PGP) (16:1/16:1)	8.0	4.5	----	----
	**Liver vs:**			
	**1^o^ Tumor**	**Brain**	**Lung**	**Spine**
N1,N8 Diacetylspermidine	42	2.8	----	2.0
1-Phenylethylamine	2.5	2.1	19.7	5.2
Pantetheine	----	----	7.3	----
	**Lung vs:**			
	**1^o^ Tumor**	**Brain**	**Liver**	**Spine**
CL^3^	----	----	----	----
Arginyl-Proline	4.6	5.3	22	3.4
DG^4^ (14:0/24:1/0:0), (16:1/22:0/0:0), (18:1/20:0/0:0)	2.9	4.7	9.7	2.5
Putreanine	3.5	3.3	7.9	3.1
	**Spine vs:**			
	**1^o^ Tumor**	**Brain**	**Liver**	**Lung**
Methionyl-Proline	71	20.8	3.1	44
Asparaginyl-Glutamate	----	26.6	16.6	----
Gentisate Aldehyde	----	26.6	16.6	----
Dityrosine	22.4	12.6	2.5	4.8
PIP2 (16:0/20:1), (16:0/22:4), & (18:0/18:1)	21.8	15.3	2.3	20.5
4-Guanidinobutanoic Acid	----	20.9	----	2.3
5-Methyldeoxycytidine	----	16.6	4.5	----
Ferrocytochrome	6.4	14.8	8.9	6.9
Pentacaboxylporphrinyl	----	10.2	5.2	5.5
SCICAR^5^	----	7.9	4.3	8.0
4-Aminobutyraldehyde	6.1	6.1	5.3	2.5
N2, N2-Dimethylguanosine	----	6.6	6.9	3.3
D-Lactaldehyde	6.2	6.6	6.1	2.5
7,8-Dihyderoneopterin	3.1	3.9	2.9	2.0

^1^1^o^ Tumor denotes primary tumor. ^2^A dash indicates that the metabolite was below the detection limit in that organ. ^3^CL denotes cardiolipins: (16:0/16:0/18:1(9Z)/18:1(9Z)), (16:0/16:0/18:1(11Z)/18:1(11Z)), (16:0/16:0/18:1(9Z)/18:1(11Z)), & (16:1 (9Z)/18:0/16:1(9Z)/18:0). ^4^DG denotes diacylglycerol. ^5^SCIAR denotes: (S)-2-[5-Amino-1-(5-phospho-D-ribosyl)imidazole-4-carboxamido]succinate.

### Raman spectroscopic differentiation of organ-specific metastatic isogenic breast cancer cell lines

**Figure 6:**
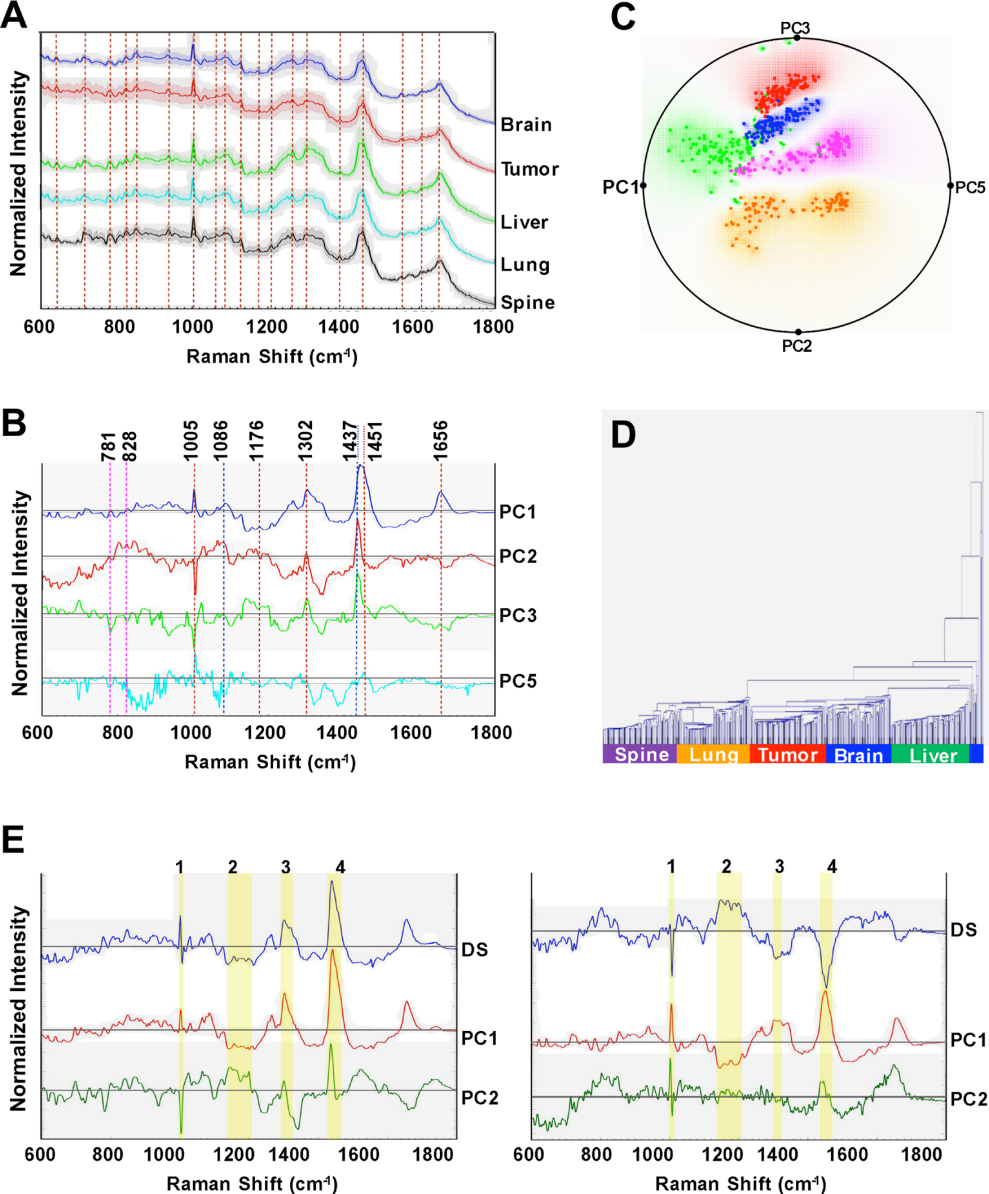
Raman spectroscopic analyses of organ-specific metastatic breast cancer cell lines reveals distinct spectral characteristics for each cell line (**A**) Representative Raman spectra acquired from brain, primary tumor (1^o^Tumor), liver, lung, and spine cell lines. The solid profile depicts the mean spectrum of each sample group and the shadow represents ±1 standard deviation. Spectra were normalized and offset for visualization. Dashed vertical lines delineate Raman shifts (cm^−1^) detailed in Table 3. (**B**) Principal component (PC) loadings for PC 1, 2, 3 and 5, for the Raman measurements are shown. Dashed vertical lines delineate prominent Raman shifts (cm^−1^) detailed in Table 3. (**C**) Radial visualization principal component scores plot, corresponding to the most discriminative PCs (PC1, 2, 3, and 5), shows the clustering of the spectral data corresponding to each organ-specific cell line, red: primary tumor, blue: brain, green: liver, orange: lung, and purple: spine. (**D**) Dendrogram of organ-specific breast cancer cell lines cluster analysis. Each color bar represents one organ-specific cell line. (**E**) Identification of informative spectral regions via PCA data exploration as exemplified by the PC loadings corresponding to the spectral dataset acquired from: primary tumor and liver (left panel) and primary tumor and spine (right panel) cell lines. The top to bottom profiles in each panel show difference spectra: (DS) between liver/primary or spine/primary spectra along with their PC1 and PC2 loadings, respectively. The highlighted yellow bars (1–4), represent the wavelength regions elucidated from the difference spectra (DS) as those with the most significant variability amongst the considered cell lines.

Mean Raman spectra (±1 standard deviation) of the metastatic isogenic breast cancer cell lines and the primary tumor cell line are shown in Figure 6A, where the spectra have been normalized and offset for visualization purposes but displayed without background correction. The Raman spectra are an aggregate expression of cellular biochemistry and structure, since the vibrational signatures inform not only on the composition of the complex biological material but also on structural states of the molecules in the specimen. The observed spectral features encode a vast amount of information of the principal constituents, [36] namely lipids, proteins, nucleic acids, carbohydrates and small molecules. Table 3 lists the assigned vibrational modes for a selection of these spectral features. Though the spectra grossly appear to have similar profiles, detailed inspection reveals subtle but discernible and reproducible shape differences, especially on removal of the broad fluorescence background [37]. Based on our previous experience in differentiation of breast tissue lesions [21], we reasoned that while the subtle distinctions between the spectra of each cell line impede the possibility of differentiation using a single feature, multivariate classification methods could enable recognition and segmentation of the cell pathology - as long as the between-class distinctions are reproducible and surpass within-class distinctions.

**Table 3:** Assignment of specific Raman spectral components to subcellular constituents

Raman Shift (cm^−1^)	DNA/RNA	Proteins	Lipids
620		C-C Twist Aromatic Ring	
643		C-C Twisting of Tyrosine	
702			Cholesterol
715	Adenine		C-N (Membrane Phospholipid Head Group)/C-N-(CH_3_)_3_
781	Cytosine/Uracil Ring Breathing		
810			Phosphodiester
828	O-P-O Stretching	Tyrosine	Phosphodiester
853		Ring Breathing of Tyrosine C-C Stretch of Proline Ring	
878			C-C-N^+^ Symmetric Stretching
938		Hydroxyproline/Proline ν(C-C) Vibration of Collagen Backbone	
1005		Phenylalanine	
1035		Collagen	
1066		Proline	Fatty Acid
1086			ν_1_CO_3_^2−^, ν_3_PO_4_^3−^, ν(C-C)Acyl Backbone
1128		C-N Stretching	
1156		C-C, C-N Stretching	
1176		C-H Bending Tyrosine	
1209		Tryptophan & Phenylalanine ν(C-C_6_H_5_)	
1241			Asymmetric Phosphate Stretching
1254		C-N in Plane Stretching	
1266		Amide III (α-Helix)/Tryptophan/Collagen	
1302		CH3/δ(CH2) Twisting, Wagging, Collagen, Amide III, & -CH2- Bending	CH3/δ(CH2) Twisting, Wagging, Phospholipids, &-CH2- Bending
1334	Nucleic Acid	CH_3_CH_2_ Wagging, Collagen	
1342		CH Deformation	
1367			*v*s (CH_3_) Phospholipids
1391		C-N Stretching in Quinoid Ring-Benzoid	
1437			CH_2_ Deformation
1451		CH_2_ Bending/CH_3_ Bending C-H Deformations	C-H Deformation
1556		Tryptophan ν(CN) & Amide II ν(C=C) Porphyrin & Tyrosine	
1605	Cytosine	Phenylalanine & Tyrosine	
1657		ν(C=O) Amide I (α-Helix) C=O Stretching of Collagen & Elastin	C=C Stretch Fatty Acids

Hence, we employed principal component analysis (PCA) to transform the dimensions of the acquired spectra into a set of linearly uncorrelated variables, i.e., principal components, along which the variation in the data is maximal (Figure 6B). This dimensional reduction step is critical to enabling sample exploration via visual assessment of similarities and differences between samples and, ultimately, in identifying the smallest possible subset of discriminatory features necessary to build a robust decision algorithm. PC1 and PC2 accounts for approximately 67% and 12% of the total variance in the dataset. In addition to homing in on the spectral features responsible for the variance between the cell lines (indicated by the dashed lines in Figure 6B), we employed the PC scores to assess the feasibility of recognizing individual cell lines based on the Raman data. Specifically, the PC scores were used to create a radial visualization plot (Figure 6C). The plot reveals the degree of clustering of the spectra recorded from the same cell line and, critically, the inter-cell line spectral variations. Together, these qualitatively suggest the presence of differential molecular constituents in the isogenic cell lines that are driven by site-specific adaptations. The Raman spectra-derived dendogram (Figure 6D), reinforces these feasibility results but also hints at the relative difficulty in separating the liver and brain cell lines based solely on the vibrational signatures. Notably, the overlap between the brain and liver Raman signatures is consistent with the metabolomics findings (Figure 5) underscoring the correspondence between the two complementary data sets.

To quantify the classification capability of Raman spectroscopy, we developed decision algorithms based on partial least squares discriminant analysis (PLS-DA) and support vector machines (SVM). The overall classification accuracy obtained for the PLS-DA-derived decision algorithm was found to be 96.8% with the classification accuracy for each cell line being in excess of 93% (Table 4). The SVM-derived decision algorithm also provides similar levels of classification performance affirming that the richness of the spectral data is the principal driver for the prediction performance. Next, we performed difference analyses across the normalized spectra obtained from pairwise comparison of cell lines to delineate the informative regions with the goal of identifying biomarkers, which would be either universal or characteristic to a specific pair of cell lines. Figure 6E exhibits two representative cases of these comparisons, namely between primary tumor and liver cell lines and between primary tumor and spine cell lines. The accompanying PC loadings were obtained from analysis of the spectral dataset constituted by the primary tumor and liver, and primary tumor and spine cell lines, respectively. By merging the difference analyses and spectroscopic basis of the PC loadings, the following informative regions were identified: 1000–1006 cm^−1^, 1136–1211 cm^−1^, 1298–1330 cm^−1^ and 1435–1470 cm^−1^.

**Table 4:** Raman spectroscopy based classification of isogenic metastatic breast cancer cell lines

Reference Identification	PLSDA ^1^algorithm	SVM ^2^algorithm	PLSDA^1^ feature-specific algorithm
	Correct Classification (%)	Correct Classification (%)	Correct Classification (%)
1^o^ Tumor^3^	99.3 (0.7)^4^	98.9 (1.1)	97.2 (2.8)
Brain	98.0 (2.0)	99.6 (0.4)	91.7 (8.3)
Liver	97.4 (2.6)	94.3 (5.7)	91.1 (8.9)
Lung	93.3 (6.7)	97.3 (2.7)	85.8 (14.2)
Spine	96.1 (3.9)	98.2 (1.9)	90.6 (9.4)

^1^Denotes Partial Least Squares Discriminant Analysis.

^2^Denotes Support Vector Machines.

^3^Denotes Primary Tumor cell line.

^4^Values in parenthesis = percentage misclassifications.

Using only the selected regions (highlighted by the yellow bars of Figure 6E), we developed a PLSDA-derived decision algorithm to reclassify all the cell lines that provided equally impressive prediction performance (Table 4) as that obtained using the full spectral analysis. Only 9.6% of the spectral information was used in this case thereby underlining the presence of specific spectral markers in the dataset.

Additionally, to ensure the robustness of these findings, we implemented a negative control study. In this case, the labels (primary tumor, brain, liver, lung and spine) were assigned in a randomized order, regardless of their actual identity. Using the acquired spectra in conjunction with these control labels, we re-derived the PLS-DA and SVM decision algorithms and used them in the same analysis protocol as detailed previously. In this situation, a low correct classification rate for each cell line was obtained with the average rate of correct classification below 20%. This underscores the robustness of the spectroscopic measurements to confounding variables and chance correlations. Collectively, these results demonstrate that Raman spectroscopy offers a reliable tool for discriminating these isogenic metastatic breast cancer cell lines on the basis of distinct organ-of-origin driven biochemical adaptations.

**Figure 7:**
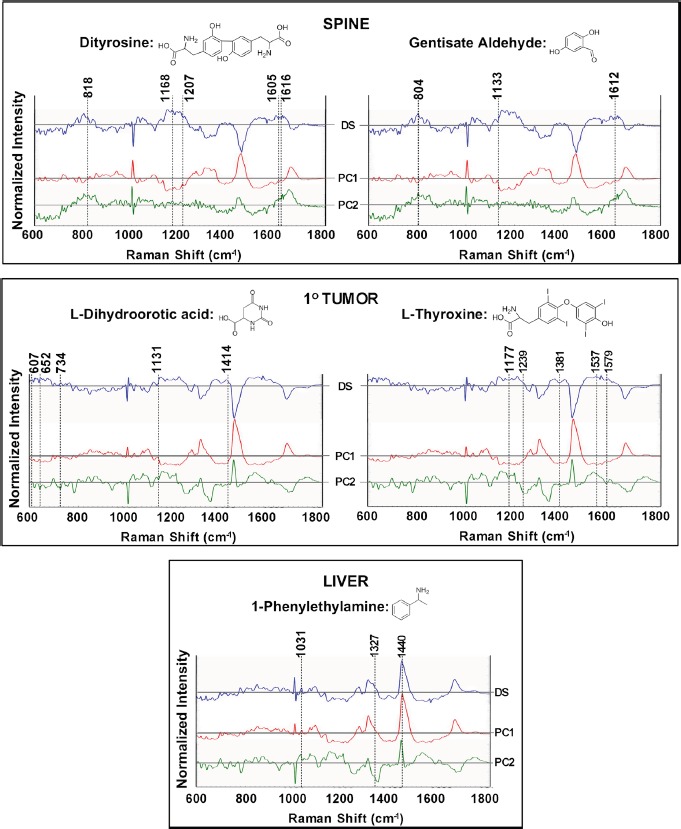
Raman spectroscopic analysis to probe the presence of cell line specific expression of molecules identified through metabolomics analysis The top panel highlights the differential expression of spectral markers in the spine cell line. The primary cell line spectrum was used as the control to calculate the difference profiles. Additionally, principal components (PC) 1 and 2, calculated from the spine and primary cell line data, are provided to capture the variance. The presence of spectral features, corresponding to the peaks of dityrosine and gentisate aldehyde, are highlighted by the dashed lines and detailed vibrational mode assignment is presented in Table 5. Similarly, the middle panel compares the Raman spectra of the primary cell line with a control group, i.e., Raman spectra acquired from the liver cell line, to illustrate the presence of features of overexpressed metabolites L-dihydroorotic acid and L-thyroxine. The bottom panel compares the Raman profiles of the liver cell line with the control group (primary) to delineate the overlap with features of 1-phenylethylamine. Profiles in blue represent the difference spectra: (DS) whereas the red and green profiles show the PC1 and PC2 loadings respectively for each chosen pair of cell lines.

We also compared Raman signatures of specific cell line pairs and tallied the spectral markers against the known Raman features of the cell line specific expressed metabolites. As show in in Figure 7-top panel, a comparison of metabolite profiles in the spine and the primary cell line reveals the overexpression of Raman-active analytes in spine, namely gentisate aldehyde and dityrosine. Similarly, complementarity of the metabolomics and Raman datasets was reinforced through detection (using liver as a control) of the overexpressed Raman-active analytes in the primary tumor: L-thyroxine and L-dihydroorotic acid (Figure 7-middle panel) and Raman-active 1-phenylethylamine in the liver (using primary tumor as a control; (Figure 7-bottom panel). Analyses of the difference spectra between cell line pairs and their corresponding PC loadings reveals the presence of subtle features at wavenumbers (scattering frequencies) where these metabolites show Raman activity (Figure 7 and Table 5).

**Table 5:** Raman shifts and associated band assignments of cell line specific metabolites

Tissue	Metabolite	Raman Shift (cm^−1^)	Band Assignment
**Spine**	**Dityrosine**	818	Tyrosine, Proline, & Hydroxyproline, ν_2_PO^2−^ stretch of nucleic acids
		1168	Tyrosine: C-H in-plane bending
		1207	Tyrosine & Phenylalanine: C-C_6_H_5_ stretching, Hydroxyproline
		1605	Tyrosine & Phenylalanine: C=C in-plane bending
		1616	Tyrosine & Tryptophan: C=C stretching mode
	**Gentisate Aldehyde**	804	νC-C, νC-O
		1133	βC-H, νC-C
		1612	νC-C, βC-H
**1^o^ Tumor^1^**	**L-Dihydroorotic Acid**	607	βC=O, βC-O_ca_, βCrg-C_ca_
		652	γNH, γOH
		734	βC=O_ca_, νC-O_ca_, νrg, βC-O_ca_
		1131	νNC, βCH1, νNC,
		1414	νNC, βNH, νrg, βrg
	**L-Thyroxine**	1177	Out-of-phase νCβ-O
		1239	In-phase νC-O, C-OH_o.o.ph_
		1381	Stretching aromatic ring
		1537	In-phase aromatic ring
		1579	Stretching aromatic ring
**Liver**	**1-Phenylethylamine**	1031	C-H in-plane bending mode, C-N stretching
		1327	CH_3_ wagging mode
		1440	CH_3_, CH deformation vibrations

^1^1^o^ Tumor denotes Primary Tumor.

## DISCUSSION

An inherent challenge within cancer research is the cataloguing of fundamental information on what is generally fatal metastatic disease within vital organs. Monitoring and treating metastatic progression remains a formidable task due to many gaps in our knowledge including: an inability to monitor the onset of successful metastatic growth along with specific differential molecular adaptations that allow for the cancer to survive and thrive within different tissue types [18, 38, 39]. Consequently, we have taken up the important consideration that metastatic cancer cells growing in visceral organs ought not to be considered simply as primary tumor implants. Rather metastatic lesions need to be understood as significantly influenced and altered by tissue-specific microenvironments that the cancer cells must adapt to [18, 38, 39]. To address this problem, we choose to characterize isogenic human breast cancer metastatic cell lines that spontaneously arose from dissemination from the primary mammary fat pad site of our mouse model.

The establishment of metastatic lesions integrated into vital visceral organs is a multistep process that includes: i) the cancer cells ability to survive as independent entities that thrive outside the normal cell-cell interactions of healthy epithelial tissues, ii) surviving harsh circulation conditions, and iii) embed and adapt to growth within microenvironments that are distinct, from a developmental as well as functional basis, from the primary tumor site. In order to mimic this process, we have generated isogenic cell lines directly from organ-specific metastatic lesions of the brain, liver, lung, and spine (bone), which are the organs most commonly affected, i.e., bone (60%), lung (34%), liver (20%), and brain (10–15%), during human metastatic breast cancer progression [25, 26]. We initially cultured these as organ explants along with a cell line from the primary tumor site, i.e., mammary fat pad, in an attempt to preserve tissue adaptation attributes acquired during adaptation to each *in vivo* microenvironment [40, 41]. In addition, these culturing conditions minimized damage as well as stresses imposed during harsher multistep single cell isolation protocols, which likely also bias cancer cell selection to subpopulations that survive the isolation procedures. This overall model is analogous to the natural course of metastatic progression found in the clinic where metastatic lesions are composed of cells that are isogenic to the primary breast cancer but also distinct at the molecular and cellular level [6–18, 38, 39].

Once adapted to plastic these new isogenic cell lines exhibited distinct phenotypic/morphological differences in growth patterns along with similarities that went across cell lines including those established from the primary tumor site. The most evident of the latter was a tendency for all cell lines to grow as complex arrays of interconnected 3D colonies with various degrees of loosely held together monolayers (Figures 1, 2, 3). The distinctions in average specific growth rates and average cell cycle rates (Figure 4 and Table 1) support the concept that the different growth patterns reflect metabolic, cell cycle, and hence, likely genetic/epigenetic differences between cell lines. Given that, in general, cytotoxic chemotherapies are more efficient in killing cycling cells, understanding differences in growth and cell cycle rates of cells in metastatic lesion will provide us with an optimum treatment strategy [42].

Attempts to distinguish between cell lines using motility assays proved inconclusive (Supplementary Figure 1) possibly due in part to the relative lack of monolayer growth exhibited to different degrees across cell lines (Figures 1, 2, 3), which is the type of lateral growth pattern that generally propagates a migration to and through a pore or into a “wound”. It is also possible that the low motility reflects a loss of this metastatic trait that is generally associated with movement out of the primary tumor, into the blood/lymph systems, and subsequent local/systemic dissemination, which may be suppressed once growth is established in a distal organ. Nevertheless, the little or no motility seen across all cell lines in the Transwell motility assay (Supplementary Figure 1) revealed that these cell lines were able to survive and grow in the low nutrient (0.1% FBS) serum conditions of the top chamber, which may reflect an adaptation to survival in a hostile microenvironment within portions of the primary tumor prior to dissemination or at the new site of growth. From a different perspective, these cell lines are ‘motile’ in up-ward growth in the form of 3D spheres and all exhibit a tendency to shed viable free-floating cells into the medium that either remained as single cells or grew as free-floating colonies that resemble mammospheres (Figure 3B). The latter trait is unusual as mammosphere growth patterns have consistently been shown to be limited to growth on non-adherent plates [43] unlike the adherent conditions used here. This trait may reflect an *in vivo* attribute of the metastatic process or adaptation to plastic. The latter seems unlikely as most available cancer cell lines adapted to growth on adherent plastic do so as monolayers with little or no “sphere” formations.

Interestingly, the colonies of brain and spine (and to a lesser extent the other cell lines as well) are interconnected and therefore, in apparent communication [44–46], by nano- or microtubes, which were observed at well over 100 μm in length (Figures 2 and 3) and at high colony densities formed complicated intricate networks between colonies (Figure 2E–2H). It appears that under these conditions the cell lines have a propensity to grow as semi-separate entities/colony arrays that require an interacting exchange of materials via these conduits [44–46]. This may be a reason for the lag-phase growth period exhibited by the metastatic brain cell line as these cells may require a relatively extensive interconnected network to support higher growth rates and shortened cell cycle times. To the best of our knowledge, at the abundance seen in our cultures, the interconnected nanotube network is a very unique characteristic along with the mammosphere formation on adherent plates.

Importantly, the metabolomics data indicates that the isogenic metastatic cell lines have diverged from the primary tumor as well as from each other (Figure 5). Table 2 shows fold increases of top metabolites, i.e., those at least 2 fold greater in each tissue-specific cell line vs all other cell lines. Although further work is needed to definitively prove whether an increase in a metabolite in a specific metastatic cell line has arisen from organ-of-origin influences, select metabolites in Table 2 can be associated with specific organs. For example, as reflected in Table 2, Neurotensin 1-10, a neurotransmitter, was found to be increased in the brain cell line and has been reported to be principally of brain origin [47] while pantetheine (vitamin B5) an intermediate in the enzyme-CoA formation pathway, which is increased in the liver cell line, is generally most abundant in liver [48]. Some classes of the tetra-acylated anionic phospholipids: cardiolipins, are only found in relatively high abundance in the lung cell line (Table 2) and cardolipins have been reported to be increased in human lung cancer [49]. Interestingly, N1, N8 diacetylspermidine has been found to be a marker of breast cancer and from our results (Table 2) it appears that it can potentially reflect metastatic progression to the liver [50]. Future studies are required to provide experimental evidence that cell line specific metabolomes contain metabolites that reflect a cell line's tissue of origin. Such work would also strive to obtain information on metabolic pathways associated with such metabolites and their potential relationships to metastatic adaptations at each site.

We also had the aim of obtaining rapid, non-destructive, and label-free profiling of these isogenic metastatic breast cancer cell lines and to this end have undertaken a Raman spectroscopy characterization approach (Figure 6). Raman spectroscopy was considered as a complementary alternative to a purely “omics” approach as the latter has some well-characterized limitations [51]. Our Raman spectroscopy-based decision algorithms showed the ability to differentiate between our isogenic cell lines with high accuracy (Table 4). These algorithms exploit subtle differences in the vibrational signatures of the molecular markers that are reflective of the multiple and complex interactions between metastatic cells and host homeostatic mechanisms. The complementary nature of these distinct analytical tools (metabolomics and Raman spectroscopy) was observed; e.g., with the general overlap that was found between the dendograms obtained from the two methods, which in both case indicated that brain and liver cell lines are closely related.

Notably, we sought more evidence of a complementarity between the two methods and found examples of Raman-active analytes, i.e., discriminating spectral markers were ascertained for cell line specific metabolites (Figure 7). As discussed earlier, the principal variations in the Raman spectra of the cell lines are largely attributable to proteins, lipids and nucleic acids however, signatures of metabolites and other small molecules are also embedded in the Raman spectra. Hence, even though fingerprinting specific metabolites through the vibrational features alone is challenging, one can infer the contributions of these metabolites towards the composite cellular biochemical status represented in the Raman data. Further probing of the high wavenumber region may provide complementary molecular insights, particularly of the lipid phenotype along with other important biochemical features [52–56].

Overall, important differences between organ-specific metastatic cell lines reflect the fact that organs differ vastly with unique attributes of metabolism, developmental programs, microenvironments, and function, all of which results in defined identities with specific growth challenges for invading cancer cells. For example, normal oxygen tension varies greatly between tissues [57]. Therefore, if one considers only this single vital nutrient change between organ types, it ought not to be surprising that a metastatic growth embedded in lung tissue with high oxygen tension would acquire different characteristics as compared to metastatic cells thriving in brain or bone at a much lower oxygen levels [57, 58].

## MATERIALS AND METHODS

### Mice

All animal handling procedures were performed in accordance with protocols approved by the Johns Hopkins University Institutional Animal Care and Use Committee and conformed to the Guide for the Care and Use of Laboratory Animals published by the NIH. Non-Diabetic severe combined immunodefcient (NOD-SCID) female mice, ages 6 to 8 weeks and initial weights of about 19–20 g, were used throughout these studies. At the end of the experiments, mice were sacrificed by administering an overdose of anesthetic [saline:ketamine:acepromazine (2:1:1)] followed by cervical dislocation.

### Cell culture and treatments

The human breast cancer cell line, MDA-MB-435, was obtained from ATCC. The MDA-MB-435 cell line was established in 1976 from a pleural effusion from an untreated 31-year-old female diagnosed with adenocarcinoma of the breast [59, 60]. MDA-MB-435 cells were authenticated at the Johns Hopkins Genetic Resource Core Facility with the short tandem repeat marker results cross checked against cell lines at the ATCC bank. Generation and characterization of the parental MDA-MB-435-tdTomato (435-tdT) cell line has been previously described [27]. All culturing was done in standard humidified incubators at 37^o^ C and 5% CO_2_. Primary tumors were initiated by injection of 2 × 10^6^ 435-tdT cells into the second thoracic mammary fat pad of 5 female NOD-SCID mice. After 13 - 15 weeks of primary tumor growth the mice were sacrificed and primary tumor, brain, liver, lungs, and, spine, were immediately excised from individual animals, dissected away from fat and muscle, and placed into sterile PBS on ice. Pieces of primary tumor, and heavily diseased lungs, and a small portion of liver with a macroscopic metastatic lesion were then immediately minced in 100 mm cell culture dishes containing 10 ml of medium within a sterile hood. All other organs/bones were inspected using fluorescence microscopy for any signs of metastatic burden, which was easily discerned as bright tdT red fluorescence (Figure 1). Areas of fluorescence along with adjacent tissue were cut away and placed into cell culture plates in sterile medium. In all cases, tissues from individual animals were cultured separately and there was no pooling of tissue samples.

All organ/bone tissue explants were initially cultured in RPMI-10%FBS supplemented with antibiotics (100 I.U./ml penicillin, 100 mg/ml streptomycin, 100 mg/ml ampicillin, and 100 mg/ml kanamycin) and, as necessary, Fungizone. The latter was often used during culturing cells out of spine as these pieces of bone, tended to float, i.e, became collagen rafts, and thus somewhat exposed at the medium to air surface, which promoted fungal growth. Medium was refreshed every 2–3 days and after two weeks of culture the medium was changed to RPMI-10%FBS supplemented with pen/strep. Over the course of 1–3 months pure red fluorescent cell cultures were obtained and the use of pen/strep in the medium was eliminated. During routine passages the medium/floating cells was first collected and the adherent colonies were then lifted off the plates by room temperature incubations in HANKS-5 mM EDTA solution for ~5–10 min with shaking and tapping by hand. Lifted cells were pooled with the collected medium/cells, centrifuged 200 xg at 21°C for 10 mins, and the supernatant (medium-EDTA) discarded. Cell pellets were then suspended in fresh medium and plated at the desired densities. It took at least 12 hr to 24 hr and at times 48 hr (generally during recovery from −80^o^ C storage) for the larger percentage of adherent cells to settle and start to grow.

### Average specific growth rate and average length of cell cycle division

Growth rate analyses were initiated by seeding 24 well plates with 10^5^ cells per well and harvesting quadruplicates of these wells every 24 hr through to the 144 hr end-point. Growth curves were generated from live cell counts obtained with a TC10 Automatic Cell Counter (Bio-Rad) in the presence of Trypan Blue. Average specific growth rates [35]: μ, were obtained from the slopes of plots of ln(N_t_/N_o_) versus time, i.e., ln(N_t_/N_o_) = μt, where N_t_is the number of cells at time ‘t’, N_o_ is the initial number of cells, and t is time. Consequently, the average length of the cell cycle was obtained from the equation: t_c_ = ln2/μ [35]. The rationale for the time intervals given in Table 1 is: The initial number of cells: N_o_, can only be obtained after the cells have had time to settle, adhere, and begin to grow, i.e., 1 day after seeding the cells, as such, day 1 in Figure 4A equals day 0 = N_o_ Figure 4B. Thus, the first time interval of 48 hrs in Figure 4B means that the number of cells: N_t_ = N_o_ + (the cells that grew between 24 and 48 hrs), i.e., each time given on the x-axis of Figure 4B represents an interval of growth that begins at day 1 of the growth curves shown in Figure 4A. Hence, average specific growth rates in Table 1 bracket times; e.g., between 24 and 72 hrs, etc (Table 1).

### Motility assay

Standard motility assays were done in 24 well Transwell^®^ plates (Costar) with 8.0 mm membrane inserts. Cells were seeded into duplicate upper chambers at a density of 10,000 cells/well in 200 ml of RPMI-0.1% FBS medium while lower chambers contained 500 ml of RPMI-5% FBS medium. Cells at the bottom surface of membranes were counted daily using a 10× objective on an inverted fluorescence microscope (Nikon Eclipse TS100) with the inherent red fluorescence of tdT as a visual marker. Two separate experiments were done and for each experiment two fields of view were counted from each well. Results indicate means ± 1 standard deviation.

### Optical microscopy

Phase contrast and fluorescence microscopy was done on a Nikon ECLIPSE TS 100 microscope (Nikon Instruments, Inc.) equipped with a Photometrics CoolSnap ES digital camera (Roper Scientific), and FITC and Texas Red filter cubes. The fluorescence light source was an X-Cite 120 Fluorescence Illumination System (Photonic Solutions, Inc.). Images were collected with NIS-Elements F3.2 software and processed with ImageJ.

### Metabolomics: principle component analysis and heat map generation

Metabolite data from all samples were acquired using Agilent 6540 Quadrupole–Time-of-Flight (Q-TOF) mass spectrometer with Agilent 1290 HPLC at the Metabolomics Facility at Johns Hopkins Medical Institutions. Data was analyzed using Agilent Mass Hunter and Agilent Mass Profiler Professional (MPP) version 13.1.1 and Agilent Qualitative and Quantitative Analysis Software packages (version 6.00) to determine the metabolic profile of each sample.

Principal component analysis (PCA) was performed to study similarities and differences among the different samples. It is a linear transformation used to describe high dimensional data [61, 62]. Expression values of metabolites and lipids were analyzed on Partek Genomics Studio 6.6 (Partek, Inc.) and used to create PCA plots. Each sphere represents a sample and each axis represents the principal components with the largest contributors being displayed. The distance between samples is inversely related to the similarity of their expression profiles, thus closely clustered samples are closely correlated. Hierarchical clustering was used to group similar expression patterns into clusters, which produced dendrograms that display the hierarchy of clustering. We clustered rows (expression values) and columns (samples) based on Euclidean distance and used average linkage method.

### Raman spectroscopy

The custom-built Raman microscope (Supplementary Figure 2) used in this work was previously reported [21]. A 785 nm Ti: Sapphire laser (3900S, Spectra-Physics), pumped by a frequency-doubled solid-state laser (Millennia 5sJ, Spectra-Physics), was used as the excitation source for the inverted microscope. The laser was focused onto the specimen using a 1.2 NA water immersion objective lens (UPLSAPO60XWIR 60X, Olympus) that also functioned to collect the backscattered signal. The collected signal was then recorded using a TE-cooled, deep depletion CCD (1340/400-EB, Princeton Instruments) following dispersion through an imaging spectrograph (HoloSpec f/1.8i, Kaiser Optical Systems). Additionally, bright field and phase contrast microscopy was performed for visualization and registration with the Raman measurements. Instead of interrogating single cells at the subcellular level, the ultimate goal of the current study is to characterize biochemical variances at the ensemble cellular level, and thus a collection of cells in pellets were investigated using point spectroscopic measurements. After replacing culture medium with PBS, cell pellets were formed by centrifugation and placed on top of the quartz coverslip for Raman measurement. Spectra (100: 10 × 10) were collected from 90μm × 90μm areas in each pellet with axial resolution of 25 μm. Raman spectra were recorded by vertical binning before averaging of 10 successive frames, each with an acquisition time of 0.3 sec, for a total collection time of 3 sec. Wavelength calibration was performed prior to spectral acquisition by acquiring spectra from 4-acetamidophenol, a Raman scatterer with well-characterized peak positions. The 600–1800 cm^−1^ fingerprint region was used for the ensuing analysis (spectral resolution of 8 cm^−1^). Cosmic ray removal was also implemented before the spectra were subjected to multivariate statistical analysis in MATLAB (Mathworks Inc.).

### Multivariate statistical analysis

While Raman spectroscopy provides a promising tool, in principle, to non-invasively probe biological specimen with high specificity, its intrinsic weak signals (especially in relation to conventional fluorescence imaging) and spectral complexity provides a substantive challenge in univariate or ratiometric quantitation of the sample constituents. Hence, to arrive at biochemical variances in isogenic cellular sublines, multivariate statistical analysis was performed on the acquired Raman spectra. By exploiting the full spectral information, as opposed to focusing on a single peak, multivariate techniques provide a robust route in extracting information both amenable and hidden from human examination.

In this study, the Raman spectra were background corrected, normalized for intensity variations, and subsequently subjected to principal component analysis (PCA). PCA is a widely used exploratory data analysis technique and employs dimension reduction to amplify the subtle differences in the recorded spectral profiles [51]. Operating without any *a priori* knowledge of the samples, PCA seeks to determine an alternate set of linearly uncorrelated coordinates, i.e., principal components (PC), such that the maximum variance in the spectral data can be explained by using only a few PCs. In particular, we employed PC scores to reveal the clustering behavior – or the lack thereof – between the metastatic breast cancer cell sublines, and the coefficient loadings to uncover the critical diagnostic variables/regions in the spectra associated with the underlying differences in the spectral data.

Additionally, to develop decision algorithms for predicting the cell type (class membership) of the spectra, partial least squares-discriminant analysis (PLS-DA) and support vector machines (SVM) were used. The former employs PLS analysis for noise reduction and variable selection and determines the maximal separation between each class by fitting a unique global model to the entire dataset. The number of loading vectors incorporated in the decision algorithm is determined by the leave-one-out cross validation procedure (LOOCV) [63].

The number of loading vectors (LV) used in the PLS-DA model was determined based on the minimal misclassification rate in a LOOCV protocol while ensuring that the spectra to LV ratio was greater than 5 to avoid problems of data sparseness. Subsequently, the dataset was split into training (70% of the spectra) and test (30%) sets to estimate the classification accuracy. This entire operation: re-splitting, training of the decision algorithm, and prediction, was performed 1000 times to obtain outcomes with well-defined statistical confidence.

Similar to PLS-DA in its supervised nature, SVM is rooted in statistical learning theory and structural risk minimization concepts and designs separating boundaries between classes by solving a constrained quadratic optimization problem. We used a radial basis function (RBF) with a Gaussian envelope to enable the separation of classes in a higher dimensional space and the optimization and kernel parameters were determined based on an automated grid search algorithm. Two different classification methods were used to confirm the validity of the results and to minimize the possibility of spurious correlations that may plague an “overfitted” decision algorithm. The output of the PLS-DA and SVM-derived decision algorithms was validated against the known class labels, i.e., the specific line of the metastatic breast cancer cellular model system. The performance of the algorithms was evaluated by determining the sensitivity and specificity using a LOOCV protocol. Similar approaches to classification of Raman spectroscopic data have been described elsewhere in the literature [22, 64].

## SUPPLEMENTARY MATERIALS FIGURES AND TABLES


