# Supplementary material for: Organ-specific isogenic metastatic breast cancer cell lines exhibit distinct Raman spectral signatures and metabolomes
Source: Oncotarget. 2017 Jan 27;8(12):20266–87. doi: 10.18632/oncotarget.14865 (PMC5386761; doi:10.18632/oncotarget.14865)
Supplement: Supplementary file 1 [file oncotarget-08-20266-s001.pdf]

Organ-specific isogenic metastatic breast cancer cell lines exhibit distinct Raman spectral signatures and metabolomes

Supplementary Materials

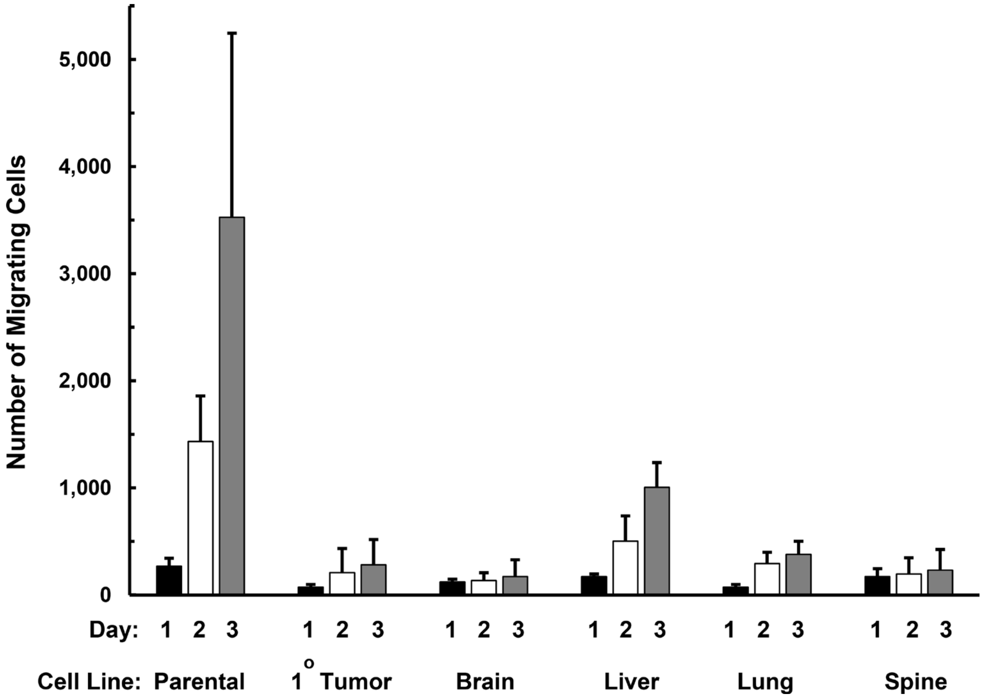

Supplementary Figure 1: Evaluation of motility through porous (8 μm) membrane inserts in standard 24 well Transwell® plates. During day two (green bars) and three (red bars), the parental cell line's motility was relatively high as compared to the isolated isogenic cell lines. On day two, the motility of the parental cell line was significantly higher than the primary tumor and all metastatic cell lines ($P < 0.05$, two tailed *t*-test) and this remained the case on day three for all cell lines except the liver cell line. By day three the liver cell line's motility was significantly higher than the primary tumor and metastatic cell lines ($P < 0.005$) but not the parental cell line. Error bars depict ± 1 SD.

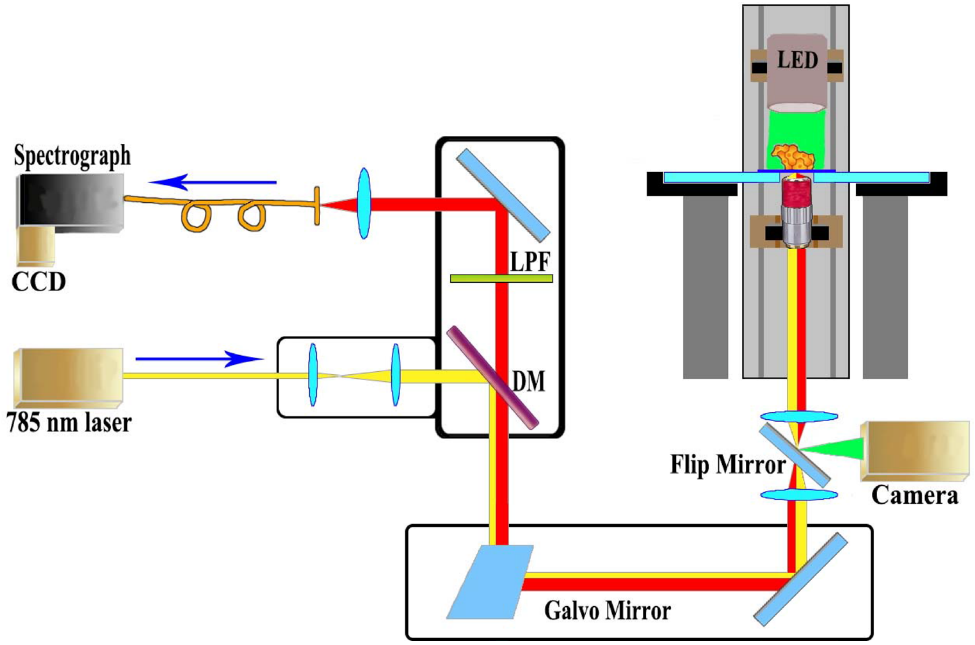

Supplementary Figure 2: Schematic illustration of high-throughput Raman spectroscopy system. The system incorporates confocal Raman, confocal reflectance (not shown here) and bright field imaging modalities for visualization and characterization of unstained live cells. LPF: Long Pass Filter; DM: Dichroic Mirror.
